# Microscopic Mechanism of Impact Sensitivity in Typical Energetic Materials: From Electronic Structure to Vibration Characteristics

**DOI:** 10.3390/ijms27072955

**Published:** 2026-03-24

**Authors:** Yuge Xiang, Jian Zhong, Ya Guo, Zhicheng Guo, Bo Jin

**Affiliations:** 1 School of National Defense, Southwest University of Science and Technology, Mianyang 621010, China; 2 State Key Laboratory of Environmental-Friendly Energy Materials, Southwest University of Science and Technology, Mianyang 621010, China; 3 Institute of Chemical Materials, China Academy of Engineering Physics, Mianyang 621999, China

**Keywords:** energetic materials, first-principle method, TATB, LLM-105

## Abstract

Energetic materials are widely used in many fields and their safety is of great concern, while the factors affecting impact sensitivity and the mechanisms of chemical bond breaking and energy transfer are still unclear. In this study, first-principles calculations were employed to elucidate the electronic and vibrational characteristics of TATB and LLM-105. Both materials are indirect semiconductors. However, LLM-105 exhibits a markedly smaller band gap and more localized nitro-antibonding conduction states, suggesting enhanced electronic excitability and potential trigger-bond activation. Phonon analysis reveals dense nitro-dominated vibrational modes in the doorway frequency region (200–700 cm^−1^), particularly in LLM-105, which may favor vibrational energy localization. In contrast, the larger band gap, delocalized conduction states, and extensive hydrogen-bonding network of TATB promote electronic and phononic energy delocalization, consistent with its lower sensitivity. These findings demonstrate that coupled electronic and vibrational effects govern stability differences in energetic materials and provide a theoretical framework for sensitivity modulation.

## 1. Introduction

Energetic materials (EMs) are a kind of material that can quickly break chemical bonds and release a large amount of energy under external stimuli. They have been widely used in the fields of defense industries such as aerospace and the military, and civil fields such as mining and construction blasting [1,2,3,4]. However, energetic materials may detonate unintentionally during transportation, storage and handling due to minor accidental external stimuli, resulting in injuries and property damage. Therefore, their safety performance has become a major concern for researchers. Safety is one of the most critical performance indicators for energetic materials, and sensitivity is commonly used to assess their safety. The sensitivity of energetic materials refers to the degree of difficulty of the explosion of energetic materials by external stimuli, which can be divided into thermal sensitivity, friction sensitivity, impact sensitivity and electrostatic sensitivity [5,6]. It is generally believed that the higher the energy of the energetic materials, the higher its sensitivity and the lower the safety [7,8]. Therefore, it is necessary to balance the relationship between energy and sensitivity when designing energetic materials.

For impact sensitivity, several theoretical frameworks have been proposed in previous research to elucidate the mechanisms underlying explosions in energetic materials, notably the hot-spot theory and the phonon up-pumping theory. The hot-spot theory posits that mechanical energy absorbed by an energetic material under external mechanical stimuli is converted into localized thermal energy at specific sites, thereby initiating combustion or detonation within the material [9]. Dlott and Fayer proposed a phonon up-pumping theory, which has been extensively employed to investigate the impact sensitivity and the rate of energy transfer in energetic materials [10]. Phonons play an important role in predicting properties related to the sensitivity of energetic materials and can be calculated and analyzed to predict the sensitivity and stability of materials, providing guidance for the design and performance optimization of materials [11]. Furthermore, Bernstein [12] identified a relationship between the phonon–molecular vibration coupling in energetic materials and the impact sensitivity of secondary explosives, proposing a method based on ab initio calculation to evaluate the impact sensitivity. Liu et al. [13] developed a theoretical model based on carrier energy transfer to evaluate the impact sensitivity of 16 energetic materials. Their findings indicated that the empirical parameter ψ, derived by incorporating factors such as the band gap, density of states, and electron mobility, exhibited a significant correlation with the H_50_ of the drop hammer experiment. Specifically, a higher ψ corresponded to reduced sensitivity of the energetic materials. This suggests that rapid electron energy transfer to adjacent molecules impedes the formation of hot spots. Conversely, Bao et al. [14,15] demonstrated that the sensitivity of energetic materials is associated with the rate of energy transfer, whereby an increased energy transfer rate corresponds to heightened sensitivity. Michalchuk et al. [16] used the phonon pumping theory to examine the influence of three polycrystalline forms of FOX-7—namely, the α, β, and γ polymorphs—on impact sensitivity. Their study predicted that variations in polymorph delamination would correspond to changes in sensitivity, specifically suggesting that increased delamination would reduce impact sensitivity. However, during experimental impact testing of the γ-FOX-7 polymorph, a phase transition to the α form was observed, which did not result in the anticipated decrease in impact sensitivity. This finding highlights the significant role of phase transitions in affecting the impact sensitivity measurements of polycrystalline energetic materials. Guo et al. [17] in their study analyzed the effect of pressure on the sensitivity of molecular crystalline energetic materials and found that the smaller the value of the band gap, the larger the integral of the number of gateway modes and the density of projected phonon states, the higher the sensitivity. Meanwhile, some researchers have calculated the phonon spectra and thermodynamic properties of energetic materials under high pressures or used molecular dynamics simulations to analyze the initiated bond breaking [18,19,20]. Despite the extensive research has been undertaken to investigate the factors influencing the impact sensitivity of energetic materials, the underlying mechanisms of chemical bond breaking and energy transfer in energetic materials under impact remain inadequately understood. Consequently, elucidating these mechanisms is essential for enhancing the safety of energetic materials and for the development of formulations that combine high energy with reduced sensitivity.

**Figure 1:**
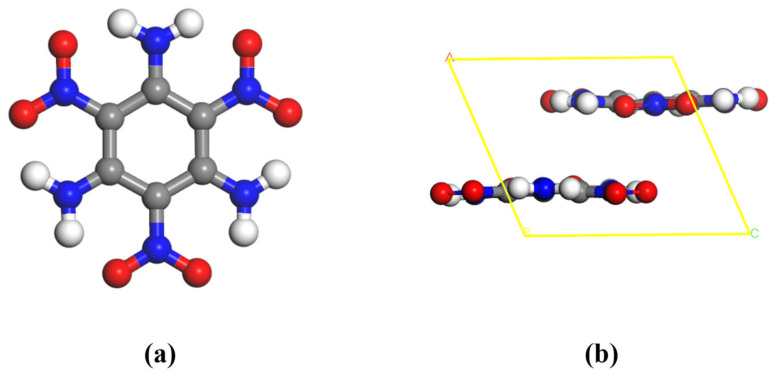
Molecular structure (**a**) and crystal structure (**b**) of TATB.

**Figure 2:**
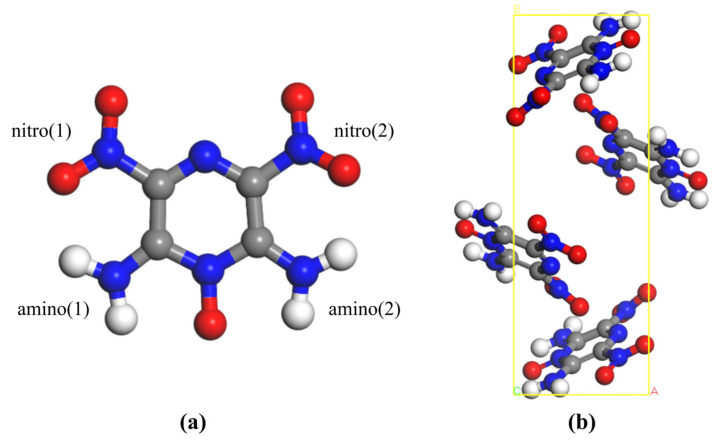
Molecular structure (**a**) and crystal structure (**b**) of LLM-105.

In recent years, 1,3,5-Triamino-2,4,6-trinitrobenzene (TATB, molecular formula C_6_H_6_N_6_O_6_) and 2,6-diamino-3,5-dinitropyrazine-1-oxide (C_4_H_4_N_6_O_5_, molecular formula C_4_H_4_N_6_O_5_) have received much attention due to their high-energy, low-sensitivity properties (used as a benchmark of insensitive high explosive). TATB has its crystal structure first determined by Candy and Larson in 1965 [21], and is known for nuclear application. Its molecular structure and crystal structure are shown in Figure 1. LLM-105 was first synthesized in Lawrence Livermore Laboratory in 1998 [22] and has a monoclinic centrosymmetric structure, and its crystal structure and cell parameters are illustrated in Figure 2. In this study, we have calculated the total density of states, projected density of states, band gap and vibrational properties of these two energetic materials by using the first nature principle and analyzed their vibrational behaviors in gateway modes, discussed the relationship between the phonon vibrational modes and the impact sensitivity of the energetic materials, and further computed the thermodynamic properties of the TATB and the LLM-105 as a function of the temperature. It is hoped that our study can provide some suggestions for future research on the development of energetic materials.

## 2. Results

### 2.1. Crystal Structure and Optimization

TATB crystallizes in the triclinic system with the space group P$\bar{1}$ and belongs to the C_i_ point group. The unit cell contains two C_6_H_6_N_6_O_6_ molecules (48 atoms). The lattice parameters of TATB are *a* = 9.01 Å, *b* = 9.03 Å, *c* = 6.81 Å, *α* = 108.59°, *β* = 91.82°, and *γ* = 119.97. LLM-105 crystallizes in the monoclinic system with the space group P2_1_/c and point group C_2h_. Each unit cell contains four C_4_H_4_N_6_O_5_ molecules (76 atoms). The corresponding lattice parameters are *a* = 5.71 Å, *b* = 15.84 Å, *c* = 8.42 Å, *α* = 90°, *β* = 101.14°, and *γ* = 90° [23].

To ensure the reliability and accuracy of the computational results, full structural optimizations were performed prior to property calculations. The optimized lattice parameters were subsequently compared with available experimental data [21,23,24,25] as shown in Table 1. The calculated unit-cell volumes (*V*) show excellent agreement with experimental values, with relative errors of 1.05% for TATB and 1.40% for LLM-105, respectively.

### 2.2. Electronic Structure

**Figure 3:**
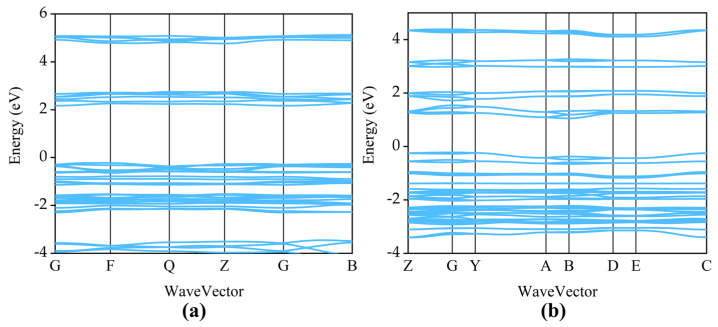
Band structure of TATB (**a**) and LLM-105 (**b**).

**Figure 4:**
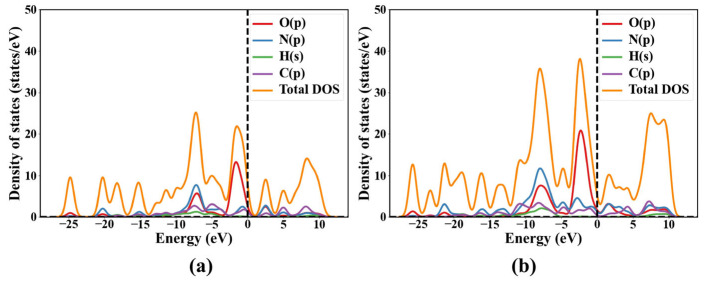
Total density of states (TDOS) and partial density of states (PDOS) for TATB (**a**) and LLM-105 (**b**).

Following structural optimization, the electronic band structures and total densities of states (DOS) of TATB and LLM-105 were calculated to elucidate their electronic characteristics. The calculated band gap of TATB is 2.3962 eV, whereas LLM-105 exhibits a narrower band gap of 1.2739 eV. For TATB, the eigenvalue of the valence band maximum (VBM) is 0.3934 eV, and that of the conduction band minimum (CBM) is 2.7896 eV. In the case of LLM-105, the VBM and CBM are located at 0.7599 eV and 2.0338 eV, respectively. Notably, for both materials, the VBM and CBM occur at different high-symmetry points in the Brillouin zone, indicating that TATB and LLM-105 are indirect band gap semiconductors. These values are in good agreement with the results reported by Qin et al. (2.366 eV) [18] for TATB and by Manaa (1.3 eV) and Xu et al. (1.547 eV) [26,27] for LLM-105. This confirming the reliability of the present computational approach. Specifically, studies have shown that the band gaps of LLM-105 and TATB calculated using the HSE06 functional are 2.227 eV and 3.6599 eV, respectively [28,29]. Compared with the HSE06 results, the PBE results do not alter the relative trend that the band gap of TATB is larger than that of LLM-105. It is well established that different exchange-correlation functionals mainly affect the quantitative values of the band gap. In particular, PBE tends to significantly underestimate the band gap due to the self-interaction error, whereas hybrid functionals such as HSE06 generally provide values closer to experimental measurements. However, the choice of functional usually does not fundamentally change the overall distribution characteristics of the band structure or the relative trends. Therefore, although the PBE functional underestimates the absolute band gap value, the distribution of the energy band structure and the relative trends discussed in this paper are still reliable. Figure 3 shows the band structures of TATB (a) and LLM-105 (b), and Figure 4 shows their total and partial density of states (TDOS and PDOS). The Fermi levels of the two energetic materials are all located in the zero value region of DOS. This feature further confirms their semiconducting nature.

### 2.3. Phonon Spectrum and Vibrational Properties

**Figure 5:**
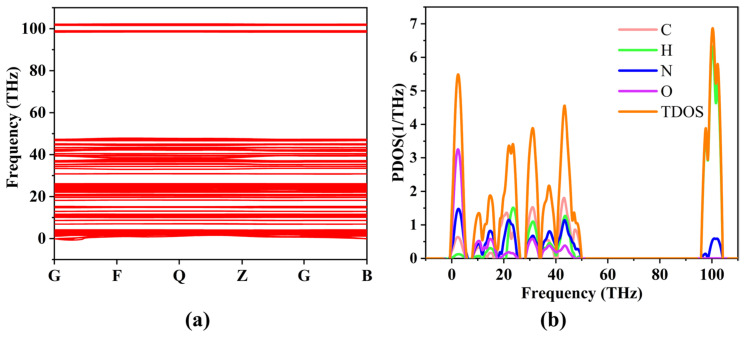
TATB phonon dispersion curve (**a**) and projected phonon density of states (**b**).

**Figure 6:**
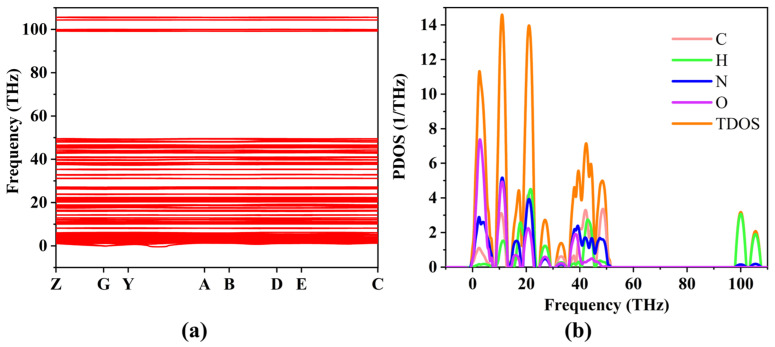
LLM-105 phonon dispersion curve (**a**) and projected phonon density of states (**b**).

The phonon dispersion relations and vibrational properties of TATB and LLM-105 were calculated using density functional perturbation theory (DFPT) based on the fully optimized crystal structures. The phonon dispersion curves, total phonon density of states, and projected phonon density of states are shown in Figure 5 and Figure 6. Minor imaginary frequencies are observed near certain high-symmetry points in both materials. Similar features have been reported in previous DFPT studies of energetic materials such as TATB, LLM-105, and HMX, and are generally attributed to numerical convergence limitations or the intrinsic softness of intermolecular lattice modes in molecular crystals [13,19,24]. Since the imaginary branches are limited and do not extend over large regions of the Brillouin zone, both crystals can be considered dynamically stable under ambient conditions.

For TATB, the primitive cell contains 48 atoms, resulting in 144 vibrational modes (M = 72A_g_ + 72A_u_), including three acoustic and 141 optical modes. Among the optical modes, 72 are Raman-active and 69 are IR-active. There are 39 vibrational modes in the 6–21 THz region. The calculated Raman vibrational frequencies show excellent agreement with previously reported theoretical results (Liu et al. [30]) and experimental measurements [31] within the 200–700 cm^−1^ range, with average deviations of 2.18% and 2.12%, respectively. For the IR-active modes, the average deviations relative to the theoretical data of Huang et al. [32] and the experimental data [33] are 3.16% and 3.57%, respectively. These small discrepancies confirm the reliability and accuracy of the present phonon calculations. In the frequency range of 8.62–21 THz (≈200–700 cm^−1^), which is often associated with doorway modes in the phonon up-pumping mechanism, TATB exhibits 37 vibrational modes (19 Raman-active and 18 IR-active). The low-frequency region (0–47.31 THz) is dominated by collective lattice and intermolecular modes involving nitro group rocking, ring deformation, and amino-group motions. The projected phonon density of states indicates that O, N, and C atoms contribute significantly in this region, reflecting strong coupling between –NO_2_ vibrations and the aromatic ring framework. In the high-frequency region (98.33–102.14 THz), the vibrational states are primarily contributed by H and N atoms, corresponding to localized amino-group stretching modes. The calculated Raman and IR frequencies within 200–700 cm^−1^ show excellent agreement with experimental and theoretical values (average deviations below ~3%), confirming the reliability of the vibrational analysis (Table 2).

For LLM-105, the unit cell contains 76 atoms, yielding 228 vibrational modes (M = 57A_g_ + 57A_u_ + 57B_g_ + 57B_u_), including 3 acoustic and 225 optical modes. Within 6–21 THz, 68 vibrational modes are identified (35 Raman-active and 34 IR-active). Comparison with previous theoretical calculations [25] and experimental measurements within the 200–700 cm^−1^ range [34,35] shows that the average deviations of the Raman frequencies are 1.37% and 1.28%, respectively, while those of the IR-active modes are 1.64% and 1.40%. These small discrepancies further confirm the high accuracy of the present phonon calculations. In the low-frequency region (0–10.51 THz), the phonon states are primarily associated with nitro-group vibrations, N-O bending modes, heterocyclic ring deformation, and partial amino motions. In contrast, the high-frequency region (99.17–105.70 THz) is mainly governed by hydrogen-related stretching modes, with dominant contributions from H atoms. The calculated Raman and IR frequencies exhibit excellent consistency with both theoretical and experimental results, with average deviations generally below 2%, further validating the computational approach (Table 3).

**Figure 7:**
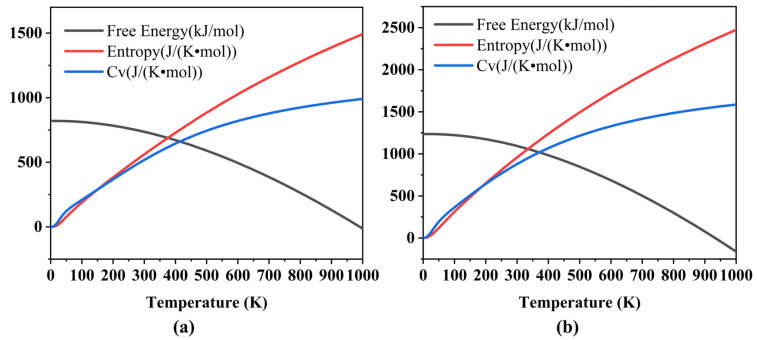
Thermodynamic properties for TATB (**a**) and LLM-105 (**b**).

The calculated temperature-dependent thermodynamic properties are presented in Figure 7. For both materials, entropy and lattice heat capacity increase monotonically with temperature, while the Helmholtz free energy decreases, consistent with harmonic lattice dynamics theory. Notably, TATB exhibits lower entropy and heat capacity values compared to LLM-105 over the investigated temperature range. Additionally, the initial free energy of TATB is lower, indicating enhanced thermodynamic stability. These differences further support the conclusion that TATB possesses a more stable lattice environment, which may contribute to its lower sensitivity under external perturbations.

## 3. Discussion

### 3.1. Electronic Structure and Its Implication for Sensitivity

According to the calculation results, the Fermi energies of TATB and LLM-105 are 0.6186 eV, 0.9788 eV, respectively. Notably, no electronic bands cross the Fermi level in any of the two energetic materials investigated, indicating the absence of metallic behavior and confirming their nonmetallic character. Furthermore, A detailed analysis of the projected density of states (PDOS) provides insight into the electronic origins of the band edges. For both TATB and LLM-105, the valence band maximum (VBM) is predominantly composed of O-2p and N-2p states, with minor contributions from C-2p orbitals. The strong participation of oxygen p-states indicates that the lone-pair electrons associated with the nitro groups play a dominant role in defining the top of the valence band. In contrast, the conduction band minimum (CBM) also primarily originates from N-2p and O-2p orbitals. However, the nature of these states differs between the two materials. In TATB, the CBM states are relatively dispersed and exhibit significant orbital hybridization, suggesting delocalized π* antibonding characteristics distributed over the molecular framework and hydrogen-bonded network. Conversely, in LLM-105, the CBM states appear more localized and show stronger contributions from nitro-related N-2p orbitals, indicating a higher degree of antibonding character associated with the -NO_2_ functional groups.

The calculated band gap of TATB (2.3962 eV) is significantly larger than that of LLM-105 (1.2739 eV), indicating that electronic excitation from the valence band to the conduction band requires higher energy in TATB. From an electronic structure perspective, a smaller band gap generally facilitates charge transfer and electronic excitation under external stimuli, which may promote population of antibonding orbitals. Given that the CBM of LLM-105 exhibits pronounced contributions from nitro-related N-2p states, electronic excitation could preferentially populate antibonding orbitals associated with the N-NO_2_ trigger bond. Such occupation may weaken the bond strength and lower the effective bond dissociation energy (BDE), thereby facilitating initial bond cleavage under mechanical or thermal stimuli. In contrast, the larger band gap and more delocalized CBM states of TATB suggest reduced electronic susceptibility to excitation-induced antibonding population. Together with its extensive intermolecular hydrogen-bonding network, this electronic stability may contribute to the well-known low sensitivity of TATB.

### 3.2. Vibrational Properties and Energy Localization

According to the phonon up-pumping theory, vibrational modes in the intermediate frequency range (200–700 cm^−1^) play an important role in energy transfer and accumulation under external stimuli. These modes facilitate coupling between low-frequency lattice vibrations and high-frequency intramolecular stretching modes, potentially leading to localized energy concentration. Both TATB and LLM-105 exhibit abundant vibrational modes in this frequency range, primarily involving -NO_2_ rocking, N-O bending, C-NO_2_ stretching, and ring deformation. These modes are directly associated with bonds commonly considered as trigger bonds in nitro-containing energetic materials, such as C-NO_2_ and N-O bonds. However, a key difference emerges in the vibrational coupling characteristics. TATB, due to its extensive hydrogen-bonding network and layered packing structure, exhibits broader phonon dispersion and stronger lattice-molecular coupling. Such features promote vibrational energy delocalization throughout the crystal lattice, potentially suppressing localized energy accumulation. In contrast, LLM-105 displays relatively more localized vibrational features in the doorway frequency range. Enhanced localization may facilitate phonon energy confinement and increase the probability of bond-selective excitation, which could contribute to its comparatively higher sensitivity.

**Figure 8:**
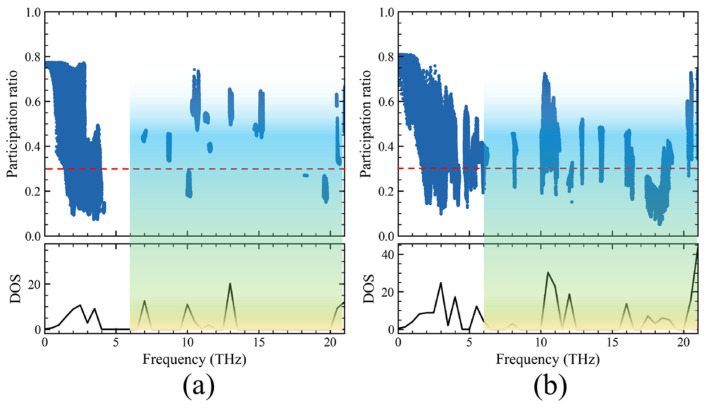
Participation ratio of phonon modes for LLM-105 (**a**) and TATB (**b**) as a function of frequency. The red dashed line represents the PR threshold of 0.3.

To quantitatively characterize the degree of vibrational localization in the doorway frequency region (6–21 THz or 200–700 cm^−1^), we further evaluated the participation ratio (PR) of the phonon modes for both TATB and LLM-105. The participation ratio provides a measure of the number of atoms contributing to a given vibrational mode and is commonly used to assess the spatial extent of vibrational excitations in molecular crystals. The calculated PR values reveal a clear difference between the two materials. In this study, a PR value of 0.3 is used as the threshold, and those with a PR value lower than 0.3 are defined as localized modes. As shown in Figure 8, the doorway modes in TATB exhibit systematically larger participation ratios, indicating that the corresponding vibrational motions involve a larger fraction of atoms in the lattice and therefore possess a more delocalized character. In contrast, the doorway modes in LLM-105 display lower PR values, suggesting stronger vibrational localization. This quantitative result is consistent with the phonon dispersion characteristics discussed above. The phonon branches of TATB in the same frequency region show larger dispersion compared with those of LLM-105, reflecting stronger intermolecular vibrational coupling. The more extensive hydrogen-bond network in TATB may therefore facilitate the redistribution of vibrational energy through the crystal lattice, whereas the comparatively weaker hydrogen-bond connectivity in LLM-105 may favor localized vibrational excitations.

**Figure 9:**
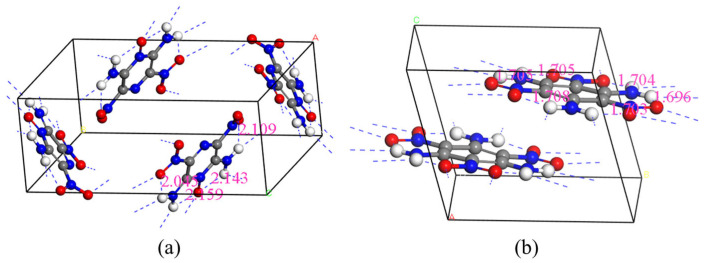
Comparison of hydrogen-bonding patterns in the crystal structures of LLM-105 (**a**) and TATB (**b**). Hydrogen bonds are indicated by dashed lines.

To further illustrate the structural differences discussed above, a schematic comparison of the hydrogen-bonding networks in TATB and LLM-105 is shown in Figure 9. In the TATB crystal, the amino groups act as hydrogen-bond donors while the nitro oxygen atoms serve as acceptors, forming an extensive intermolecular hydrogen-bond network that stabilizes the layered molecular packing. The hydrogen bond length is around 1.7 Å. This highly interconnected hydrogen-bond topology enhances intermolecular coupling and can facilitate the redistribution of electronic and vibrational energy within the crystal lattice.

In contrast, although hydrogen bonding is also present in LLM-105, the overall network is comparatively less extensive and less interconnected than that in TATB. The hydrogen bond length is around 2.1 Å, which is longer than that of the TATB hydrogen bond. As a result, intermolecular interactions are relatively weaker, which is consistent with the more localized electronic states and vibrational doorway modes identified in the present calculations. The difference in hydrogen-bond connectivity may therefore contribute to the distinct mechanisms of energy transfer and stability observed for the two energetic materials.

Therefore, the vibrational analysis complements the electronic structure results: while the smaller band gap of LLM-105 facilitates electronic excitation into antibonding orbitals associated with the N-NO_2_ bond, its vibrational characteristics may also promote more efficient energy localization. In TATB, both electronic stability (larger band gap and delocalized CBM states) and vibrational energy dispersion act cooperatively to enhance structural robustness.

## 4. Materials and Methods

In this study, first-principles calculations were performed to systematically investigate the electronic and vibrational properties of the energetic materials. The initial crystal and molecular structures were obtained from the Cambridge Crystallographic Data Centre (CCDC). Density functional theory (DFT) calculations were carried out using the Vienna Ab initio Simulation Package (VASP 5.4.4) to optimize the crystal structures of the two energetic materials and to evaluate their electronic properties [36]. The exchange–correlation interactions were described using the generalized gradient approximation (GGA) with the Perdew–Burke–Ernzerhof (PBE) functional. To accurately account for long-range dispersion interactions that are critical in molecular crystals, van der Waals corrections were incorporated using the Grimme DFT-D3 scheme [37]. The interaction between the ion cores and valence electrons was treated using the projector augmented-wave (PAW) method within the plane-wave basis framework. A plane-wave cutoff energy of 830 eV was employed to ensure sufficient convergence accuracy. The electronic self-consistent field (SCF) calculations were converged to an energy criterion of 1 × 10^−7^ eV/atom during structural optimization. Brillouin zone integrations were performed using Monkhorst–Pack k-point meshes of 2 × 2 × 2 for TATB and 3 × 1 × 2 for LLM-105, respectively. Phonon dispersion relations were calculated using the Phonopy 2.38.2 package based on density functional perturbation theory (DFPT) to evaluate the dynamical stability of the energetic materials [38,39]. Supercells of 2 × 2 × 2 (TATB) and 2 × 1 × 2 (LLM-105) were employed to ensure reliable evaluation of interatomic force constants while maintaining consistent k-point sampling. Thermodynamic quantities including Helmholtz free energy, entropy, and constant-volume heat capacity were derived within the harmonic approximation.

## 5. Conclusions

In this work, first-principles calculations were used to compare the electronic structure and lattice dynamics of TATB and LLM-105. The optimized structures are in good agreement with experimental data, confirming the reliability of the calculations. Both materials are indirect band-gap semiconductors, but LLM-105 has a much smaller band gap and more localized nitro-related antibonding conduction states, indicating a greater propensity for excitation-induced trigger-bond activation. By contrast, TATB shows a larger band gap and more delocalized conduction states, consistent with lower electronic susceptibility. Phonon analysis further shows that LLM-105 possesses denser nitro-dominated doorway modes and stronger vibrational localization in the 200–700 cm^−1^ region, which may favor energy confinement and bond-selective excitation. In contrast, the broader phonon dispersion, higher participation ratios, and more extensive hydrogen-bonding network of TATB facilitate electronic and vibrational energy delocalization and thereby enhance structural robustness.

Overall, the results indicate that impact sensitivity is governed by coupled electronic excitability and vibrational energy localization. This dual electronic–vibrational model can therefore serve as a useful predictive tool for the design of new insensitive energetic materials: candidates with larger band gaps, less localized antibonding conduction states, weaker doorway-mode localization, and stronger intermolecular energy-delocalization pathways are expected to exhibit lower sensitivity.

## Figures and Tables

**Table 1:** Calculated crystal structure parameters and experimental values for TATB and LLM-105 [21,23,24,25].

	*a* (Å)	*b* (Å)	*c* (Å)	*α* (°)	*β* (°)	*γ* (°)	*V* (Å^3^)	References
TATB	9.098	9.114	6.767	108.785	91.780	120.011	447.169	This work
	9.01	9.028	6.812	108.58	91.82	119.97	442.524	Expt. [21]
	9.065	9.081	6.762	108.72	91.92	119.98	443.76	Expt. [24]
LLM-105	5.761	15.909	8.422	90	101.116	90	757.367	This work
	5.71	15.84	8.42	90	101.14	90	746.912	Expt. [23]
	5.774	15.696	8.488	90	101.251	90	754.5	Expt. [25]

**Table 2:** Raman and IR vibrational frequencies of TATB and their assignments.

	Raman Frequencies (cm^−1^)			Assignments
Mode	This Work	Liu et al. [30]	Exp. [31]	This Work
Q26	229.49			Ring distorsion + NO_2_ rock + NH_2_ rock
Q27	287.53	292	296	NO_2_ rock + NH_2_ rock
Q29	288.20	295	297	NO_2_ rock + NH_2_ rock
Q30	288.53			NO_2_ rock + NH_2_ rock
Q32	337.90	312	332	Ring distorsion
Q33	338.90	318	334	Ring distorsion
Q36	359.58	370	369	NH_2_ rock + NO_2_ rock
Q38	361.25	371	371	Ring deformation + NH_2_ rock + NO_2_ rock
Q42	386.93	391	391	C-NO_2_ stretch
Q44	431.97	436	445	NH_2_ rock
Q46	432.63	438	449	NH_2_ rock + NO_2_ rock
Q49	510.02	520	524	NH_2_ rock + NO_2_ rock
Q50	510.35	521		NH_2_ rock + NO_2_ rock
Q53	606.42			Ring distorsion + NH_2_ stretch
Q54	606.42	611		Ring distorsion + NH_2_ stretch
Q57	654.79	656		Ring distorsion + NO_2_ waggle
Q58	658.12	660		Ring distorsion + NO_2_ waggle
Q61	687.14	665	683	Ring deformation + NO_2_ waggle
Q62	687.14	691	700	Ring deformation + NO_2_ waggle
Q63	699.82	704	704	Ring distorsion + NO_2_ scissor + C-NO_2_ stretch
	**IR Frequencies (cm^−1^)**			**Assignments**
**Mode**	**This Work**	**Huang et al. [32]**	**Exp. [33]**	**This Work**
Q25	229.16			Ring distorsion + NO_2_ rock + NH_2_ rock
Q28	288.20	294.07		NO_2_ rock
Q31	337.57			Ring distorsion
Q34	340.24			Ring distorsion
Q35	358.58	353.80		Ring deformation + NO_2_ rock + NH_2_ rock
Q37	360.25			NH_2_ rock + NO_2_ rock
Q39	380.93			NO_2_ rock
Q40	381.93			NO_2_ rock
Q41	386.60			C-NO_2_ stretch
Q43	431.30			NH_2_ rock + NO_2_ rock
Q45	431.97	458.57	447.96	NH_2_ rock + NO_2_ rock
Q47	489.34			NH_2_ rock + NO_2_ rock
Q48	490.01			NH_2_ rock + NO_2_ rock
Q51	510.35			NH_2_ rock + NO_2_ rock
Q52	510.69	539.32		NH_2_ rock + NO_2_ rock
Q55	653.12	644.32		Ring distorsion + NO_2_ waggle
Q56	653.12			Ring distorsion + NO_2_ waggle
Q59	681.81			Ring twist + NH_2_ rock
Q60	682.14			Ring twist + NH_2_ rock

**Table 3:** Raman and IR vibrational frequencies of LLM-105 and their assignments.

	Raman Frequencies (cm^−1^)			Assignments
Mode	This Work	Yuan et al. [25]	Exp. [34]	This Work
Q48	208.48	205.20		NO_2_ (1) & (2) rock
Q49	267.85	264.37	268.56	NO_2_ (1) & (2) rock + N-O i.p.bend + NH_2_ (2) rock
Q51	275.52	271.97		NO_2_ (1) & (2) rock + N-O i.p.bend + NH_2_ (2) rock
Q55	336.90	331.26		Ring deformation + N-O o.p.bend
Q56	338.90	334.12		Ring deformation + N-O o.p.bend
Q59	344.57	340.29		Ring deformation + NO_2_ rock
Q60	345.91	342.14		Ring deformation + NO_2_ rock
Q62	350.58	346.13		Ring deformation + C-NH_2_ str
Q63	350.58	348.12	350.67	Ring deformation + C-NH_2_ (2) o.p.bend
Q66	360.25	353.58		NO_2_ (1) & (2) rock + C-NH_2_ (1) & (2) i.p.bend + C-NO_2_ (1) str
Q68	362.58	356.04		NO_2_ (1) & (2) rock + C-NO_2_ (1) str + C-NH_2_ (1) i.p.bend
Q71	373.59	363.67		NO_2_ (1) & (2) rock + NH_2_ (1) & (2) rock + C-NH_2_ (1) i.p.bend
Q72	376.93	365.89		NO_2_ (1) rock + NH_2_ (1) rock+ C-NH_2_ (1) i.p.bend
Q75	402.28	393.09		NH_2_ (2) rock + N-O i.p.bend + C-NH_2_ (2) i.p.bend
Q76	406.61	398.09	409.98	NH_2_ (2) rock + N-O i.p.bend + C-NH_2_ (2) i.p.bend
Q77	427.96	424.10	428.22	NO_2_ (1) rock + N-O i.p.bend
Q78	428.63	424.19		NO_2_ (1) rock + C-NO_2_ (1) i.p.bend + N-O i.p.bend + C-NH_2_ (1) str
Q82	474.66	470.90	478.40	NO_2_ (1) & (2) rock + N-O str
Q84	478.33	474.23		NO_2_ (1) & (2) rock + N-O str
Q85	533.37	529.03	537.71	Ring deformation + N-O str + C-NH_2_ str
Q86	535.04	529.65		Ring deformation + N-O str + C-NH_2_ str
Q89	545.71	533.16		Ring deformation
Q92	548.71	538.58		Ring deformation
Q93	587.41	575.15	555.96	NH_2_ (1) & (2) waggle
Q96	608.42			NH_2_ (1) waggle + NH_2_ (2) twist
Q99	622.10			Ring deformation + NH_2_ (2) twist + NH_2_ (1) waggle
Q100	624.77			NH_2_ (1) waggle
Q102	628.10	617.02	633.51	Ring deformation + C-NH_2_ o.p.bend
Q105	675.47	670.33		Ring deformation + C-NO_2_ (1) & (2) o.p.bend + C-NH_2_ (2) o.p.bend
Q106	677.80	672.21		Ring deformation + C-NO_2_ (1) & (2) o.p.bend + C-NH_2_ (1) o.p.bend + N-O o.p.bend
Q109	683.47	678.99		NO_2_ (1) & (2) scissor + C-NO_2_ str
Q110	684.81	680.09		NO_2_ (1) & (2) scissor + C-NO_2_ str
Q112	686.47	681.71	701.94	NO_2_ (1) & (2) scissor + C-NO_2_ str
Q113	699.82	696.59		Ring deformation + C-NO_2_ (1) o.p.bend + C-NH_2_ (2) o.p.bend
Q114	700.15	697.43		Ring deformation + C-NO_2_ (1) o.p.bend + C-NH_2_ (2) o.p.bend
	**IR Frequencies (cm^−1^)**			**Assignments**
**Mode**	**This Work**	**Yuan et al. [25]**	**Exp. [35]**	**This Work**
Q47	206.14	202.64		NO_2_ (1) & (2) rock
Q50	271.19	267.31		NO_2_ (1) & (2) rock + N-O i.p.bend + NH_2_ (2) rock
Q52	277.53	273.71		NO_2_ (1) & (2) rock + N-O i.p.bend + NH_2_ (2) rock
Q53	335.23	328.38		Ring deformation + N-O o.p.bend
Q54	336.23	330.75		Ring deformation + N-O o.p.bend
Q57	343.57	339.50		NO_2_ (2) scissor + NO_2_ (1) rock + C-NO_2_ (2) str + C-NH_2_ (1) str
Q58	343.57	339.95		NO_2_ (2) scissor + NO_2_ (1) rock + C-NO_2_ (2) str + C-NH_2_ (1) str
Q61	349.24	344.78		Ring deformation + NO_2_ rock
Q64	351.91	348.55		NO_2_ (1) & (2) rock
Q65	357.91	350.63		NH_2_ (1) & (2) rock + C-NH_2_ (1) & (2) i.p.bend
Q67	361.58	354.70		NO_2_ (2) rock + C-NH_2_ (1) & (2) i.p.bend + NH_2_ (1)) rock
Q69	365.92	360.58		NO_2_ (1) & (2) rock + C-NH_2_ (1) i.p.bend
Q70	367.92	362.01		NO_2_ (1) & (2) rock + C-NH_2_ (1) i.p.bend + N-O i.p.bend
Q73	395.94	387.67		NH_2_ (2) rock + N-O i.p.bend + C-NH_2_ (2) i.p.bend
Q74	400.94	392.81		NH_2_ (2) rock + N-O i.p.bend + C-NH_2_ (2) i.p.bend
Q79	429.96	426.10		NO_2_ (1) rock + N-O i.p.bend + C-NH_2_ (1) str
Q80	430.96	426.69		NO_2_ (1) rock + N-O i.p.bend
Q81	472.33	468.41		NO_2_ (1) & (2) rock + N-O str
Q83	475.33	471.29		NO_2_ (1) & (2) rock + N-O str
Q87	537.37	530.68		Ring deformation + N-O str + C-NH_2_ str
Q88	537.71	532.01		Ring deformation + N-O str + C-NH_2_ str
Q90	547.05	533.26		Ring deformation
Q91	548.05	536.41		Ring deformation
Q94	589.74	575.92		NH_2_ (1) waggle
Q95	589.74	577.04		NH_2_ (1) waggle + C-NH_2_ (1) o.p.bend
Q97	608.75	588.42		NH_2_ (2) twist
Q98	609.76	589.08		NH_2_ (2) twist
Q101	626.77	616.41		Ring deformation + C-NH_2_ (2) o.p.bend
Q103	629.44	618.56	619.00	Ring deformation + C-NH_2_ (1) & (2) o.p.bend + NH_2_ (2) twist
Q104	629.77	618.67	632.98	Ring deformation + C-NH_2_ (1) & (2) o.p.bend + NH_2_ (1) & (2) twist
Q107	678.14	672.81		Ring deformation + C-NO_2_ (1) & (2) o.p.bend + C-NH_2_ (1) o.p.bend + N-O o.p.bend
Q108	680.14	674.09	666.68	Ring deformation + C-NO_2_ (1) & (2) o.p.bend + C-NH_2_ (1) o.p.bend + N-O o.p.bend
Q111	685.81	680.78		NO_2_ (1) & (2) scissor + C-NO_2_ (1) str

Note: i.p.bend: in plane bending; o.p.bend: out plane bending; str: stretch.

## Data Availability

The data presented in this study are available on request from the corresponding author due to (specify the reason for the restriction).
